# Epigenetic analyses in forensic medicine: future and challenges

**DOI:** 10.1007/s00414-024-03165-8

**Published:** 2024-01-20

**Authors:** Maria Carla Gerra, Cristina Dallabona, Rossana Cecchi

**Affiliations:** 1 Department of Chemistry, Life Sciences, and Environmental Sustainability, University of Parma, Parco Area Delle Scienze 11a, Viale Delle Scienze 11a, 43124 Parma, PR Italy; 2 Department of Medicine and Surgery, University of Parma, Via Antonio Gramsci 14, 43126 Parma, PR Italy

**Keywords:** Forensic epigenetics, miRNA, DNA methylation, Post-mortem stability, Histone modifications, Tissue-specificity

## Abstract

The possibility of using epigenetics in forensic investigation has gradually risen over the last few years. Epigenetic changes with their dynamic nature can either be inherited or accumulated throughout a lifetime and be reversible, prompting investigation of their use across various fields. In forensic sciences, multiple applications have been proposed, such as the discrimination of monozygotic twins, identifying the source of a biological trace left at a crime scene, age prediction, determination of body fluids and tissues, human behavior association, wound healing progression, and determination of the post-mortem interval (PMI). Despite all these applications, not all the studies considered the impact of PMI and post-sampling effects on the epigenetic modifications and the tissue-specificity of the epigenetic marks.

This review aims to highlight the substantial forensic significance that epigenetics could support in various forensic investigations. First, basic concepts in epigenetics, describing the main epigenetic modifications and their functions, in particular, DNA methylation, histone modifications, and non-coding RNA, with a particular focus on forensic applications, were covered. For each epigenetic marker, post-mortem stability and tissue-specificity, factors that should be carefully considered in the study of epigenetic biomarkers in the forensic context, have been discussed. The advantages and limitations of using post-mortem tissues have been also addressed, proposing directions for these innovative strategies to analyze forensic specimens.

## Introduction

Genetic analysis has been widely employed in the last decades on a variety of biological tissues to uncover individuals’ DNA profiles and thus to answer questions of interest to a court of law. However, in the last years, attention has grown to a new area of genetics called epigenetics. Epigenetics is the study of molecular processes that influence variable gene expression patterns on the basis of a DNA sequence that is always constant and includes DNA methylation, histone modification, chromatin remodeling, and non-coding RNA.

One of the most intriguing aspects is that the environment can influence the epigenetic signatures. Environmental exposures and our behaviors, including stress, lifestyle, drugs, and diet, constantly communicate signals to our cells that often shape epigenetic modifications to adapt to a specific situation, through changes in gene expression, without affecting DNA sequence. When the original process that induces the modification is over, the modifications might still accumulate throughout a lifetime and even be passed from parents to offspring, a phenomenon referred to as epigenetic inheritance. In fact, increasing evidence suggests that epigenetic information can not only be mitotically inherited but also meiotically transmitted in several organisms, including humans. However, evidence of transgenerational inheritance in humans via germline in the absence of any direct exposure to the driving external stimulus remains controversial [1]. Alternatively, epigenetic marks can be transient, and, unlike genetic variants, they can be reversible [2]. This has been particularly attractive in cancer research in the past years because the enzymes adding or removing the epigenetic tags have become targets for new drugs’ development to try to restore the original setting of the genes through epigenetic editing [3]. The evidence of epigenetic changes influencing factors, such as the environment, substance use disorders, and past life experiences [4], which in turn can affect the behavior of individuals, might be of great interest in the forensic field, given their potential impact on the judicial evaluation of the role of a criminal or a crime victim. The reversibility of epigenetic modifications, as well as their hereditary transmission, opens further areas of involvement for forensic medicine, which is destined to become increasingly interested in these issues. It also presents a wide field of research for forensic psychiatry in criminal management.

Among the most studied environmental exposures, it was largely demonstrated that compulsive drug use leading to addicted states implies altered plasticity and physiology of the brain, which can be partly driven by epigenetic phenomena. This affects the acute response to drugs and the development of addiction [5]. For example, regulation of histone marks and DNA methylation by cocaine, cannabis, methamphetamine, or morphine has been correlated with changes in gene expression in addiction models and humans [5–7]. Chronic stress was also demonstrated to cause long-lasting epigenetic changes, triggering mental or psychiatric disorders [8]. Adverse experiences, such as physical injury, natural disaster, bullying, and childhood maltreatment, involve long-term epigenetic modifications and highlight the complex crosstalk between the environment and our genome across development [9–11]. These issues always involve forensic medicine and forensic sciences, so, understandably, the future holds great research opportunities in this field.

In light of the environmental effect, forensic epigenetics, applying epigenetics techniques, might help to address a wide range of other questions of interest to a court of law, obtaining information from a crime scene stain and addressing challenges to the evidence that can be made in the court [12]. Epigenetic analyses have already been proposed for some forensic applications: differential DNA methylation among tissues and individuals, for example, has been used to determine the tissue type of a human biological trace, for the age estimation of an unknown trace donor, and to differentiate between monozygotic twins [13]. Non-coding RNAs (ncRNAs) among which microRNA (miRNA) in post-mortem tissues seem to represent an excellent tool to evaluate the elapsed time since death or the post-mortem interval (PMI), because of their evidenced stability and tissue specificity [14, 15]. Recently, the detection of the histone mark H3K4me3 (the trimethylation at the lysine 4 in histone H3) in chromatin has been proposed to reflect transcriptional changes in cases with substance use disorders and neurological deficits [16]. Certain authors have also proposed the concept of a new research area known as behavioral epigenetics. For instance, two studies suggested that DNA methylation could potentially serve as a marker of aggressive behavior, specifically within the glucocorticoid receptor genes *NR3C1* [17] and *NR3C2* [18]. However, it should be recognized that the epigenome is dynamic and modulated by internal and external factors and often requires complex data interpretation. In addition, the application of epigenetic techniques for predicting decision-making processes or criminal behaviors raises significant legal and ethical considerations [19]. For this reason, experts in the field, especially forensic pathologists and legal experts, are encouraged to deepen the study of epigenetics with their rigorous methods. This will help determine which information derived from epigenetics can potentially prove valuable within the context of the justice system.

The present review aims to provide a general overview to forensic scientists or forensic pathologists, who are not familiar with epigenetics, on the role of epigenetic modifications and their possible application in forensics, trying to stimulate the interest of young scientists in this new and promising field of research. Moreover, while the number of epigenetic studies in forensic medicine is steadily increasing, it is fundamental to consider the impact of PMI and post-sampling effects on epigenetic modifications such as DNA methylation, histone modifications, and non-coding RNA. It is also important to acknowledge that epigenetic marks exhibit tissue-specific patterns of expression [20]. The role and function of epigenetic modifications have been summarized, with a particular focus on their post-mortem stability and tissue specificity, which are of primary importance to forensic scientists. This information might clarify the limitations and the advantages of epigenetic analyses in forensic research, assisting researchers in choosing the most suitable marker for their studies.

## Materials and methods

Peer-reviewed articles related to the post-mortem stability and tissue-specificity of the epigenetic modifications were comprehensively selected, using terms related to forensic sciences, DNA methylation, histone modifications, chromatin, and non-coding RNA. The literature review was performed in the following international databases: PubMed, MEDLINE, Google Scholar, Embase, and Scopus, considering publications up to December 2022. A first screening of the articles was completed by reading their headlines and abstracts to ensure that the topic and content were relevant and of clear forensic interest. This preliminary step was conducted according to the inclusion criteria: publications in the English language only or availability of English abstract, starting biological material constituted by human samples, and range of publication time 2005–2022. Subsequently, a more in-depth screening based on inclusion criteria and quality assessment allowed to extract a series of data to build Table 1 and 2 of the present review. In detail, the data included are sample compositions and size, analyzed tissues, molecular targets, design and methods employed, data analysis and results, authors and year of publication, and PMID. Potentially relevant studies that did not appear in the main search were also identified from the References of other articles and consultation with experts in the field.

**Table 1:** Studies on the epigenetic modifications’ stability in post-mortem human tissues

Tissues and participants	Epigenetic mark	Methods	Findings	Authors	PMID
6 frozen postmortem brain regions from 6 subjects	DNA methylation at specific sites of *SLC6A4* and *ALDH2* genes	Bisulfite sequencing	PMI relates to the amount of degradation and methylation variance. Blood and brain sample methylation varied only in small degrees from the global mean, pointing toward the translational capacities	Rhein et al. [1]	26,042,147
Buccal swabs taken postmortem during different stages of decomposition and PMI of 1–42 days from 73 decedents	Age-associated CpG-1 site of *PDE4C* gene (upstream of cg17861230)	Bisulfite pyrosequencing	Plotting the chronological age vs the degree of *PDE4C* CpG-1 methylation, no relevant influence of the state of decomposition was found. DNA yields of swabs were unexpectedly high in the postmortem cases with signs of decomposition	Koop et al. [2]	32,632,799
14 formalin-fixed paraffin-embedded tissue blocks after postmortem delays of 0, 24, 48, and 72 h from 14 autopsy cases	DNA cytosine modifications and histone modifications	Human brain tissue microarray	The epigenetic modifications were stable up to 4 days postmortem: DNA cytosine modifications and histone methylation resulted stable; subtle changes were detected in histone acetylation at 4 to 5 days postmortem	Jarmasz et al. [3]	30,635,019
Frozen postmortem prefrontal cortex from 16 normal adult subjects	H3K4me3 and H3K27me3	Chromatin immunoprecipitation	The nucleosomal organization of genomic DNA is preserved in postmortem tissue for at least 30 h after death; differences in histone methylation levels between various genomic loci are maintained in postmortem brain even after prolonged autolysis	Huang et al. [4]	16,574,239
Frozen human prefrontal cortex brain specimens from 6 cases	H3K27Ac and H3K4me3	Chromatin immunoprecipitation (ChIP-Seq)	H3K27Ac or RNA polymerase not consistently detected, while the enhancer H3K4me3 histone modification was abundant and stable up to the 72 h postmortem	Basova et al. [5]	33,805,201
Frozen brain tissues (cortex, cerebellum and brain stem) from 12 cases	Methyltransferase and acetyltransferase activities	Enzyme Activity Kit	Methyltransferase and acetyltransferase activities relatively preserved with PMI and storage duration. No direct influence of postmortem variables on the RNA integrity with PMI of 5 h	Monoranu et al. [6]	20,652,834
18 histological forensic formalin-fixed paraffin-embedded samples from 4 individuals, collected at autopsy at different PMI (18, 65, 72, 96 h)	Transcripts of beta-actin, GAPDH, histone H3 genes, the small RNA RNU6-2 and miR21	RT-qPCR	RNA transcripts are similarly degraded in all the postmortem organ tissues. Small RNA molecules (RNU6-2 and miR21) are stable even in compromised tissues at different PMI	Muciaccia et al. [7]	25,135,750
Cardiac tissue collected within 1 week of the patient’s death and either frozen (19 samples) or fixed in formalin for up to 3 years (36 samples)	hsa-miR1, hsa-miR133a-3p, hsa-miR208b, hsa-miR499a-5p. Controls: U6 snRNA, U47, RNU6B, miR191, miR93, miR26b	TaqMan MicroRNA Reverse Transcription kit	Endogenous controls for miRNA quantification have a crucial importance; miRNA are more appropriate than other classes of small ncRNA in the postmortem analysis; miR191 and miR26b less influenced by the PMI or the long-term fixation	Kakimoto et al. [8]	26,046,358
Frozen skin specimens in autopsy cases of death due to hanging, paraffin-embedded skin samples for validation	Panel of miRNA	miScript® miRNA PCR Array	No significant differences between frozen and formalin-frozen samples: confirmed the full applicability of the analyses to the formalin-fixed samples	Neri et al. [9]	31,882,882
61 tissue biopsies of 24 different organs from 2 male bodies	2007 miRNA of miRBase V19 (first corpse) and 1997 human miRNA of miRBAse v21 (second corpse)	SurePrint 8 × 60 K Human V19 miRNA microarrays Agilent and tissue specificity index calculation	A storage time between 1 and 14 days at 4 °C has a limited influence on the overall miRNA tissue pattern	Ludwig et al. [10]	26,921,406
Fresh cortical tissues from 20 patients subjected to epilepsy surgery; 4 postmortem brain samples as controls (average PMI 29 h, ± 2.6 h)	Gene expression of 15,655 genes	RNA sequencing and microarrays	Remarkable differences detected in transcriptional patterns between fresh and postmortem brain	Dachet et al. [11]	33,758,256
2016 high-quality postmortem samples from 15 tissues from 316 donors (PMI = 1–27 h)	Gene expression	RNA sequencing	mRNA degradation is associated to PMI and it is tissue-specific, gene-specific, and even genotype-dependent	Zhu et al. [12]	28,710,439
Brain tissues from 1068 donors	Gene expression of *PAK2*; *SERBP1*; *TUBA4A*; *ACO1*; *NAPA*; *PRDX5*; *ETFB*; *GSTM5*; *MCTS1*; and* ACTB*	TaqMan Gene Expression Assays	Average RIN value was independent of the PMI, up to at least 36 h: RIN values is more critical than PMI for determining suitability of tissues for molecular biological studies; tissues stored up to 23 years at − 80 °C yielded high-quality RNA	White et al. [13]	29,498,539
12 skin samples from 6 patients (6 samples at 24 °C and 6 at 40 °C for 5 days)	Gene expression of the skin-specific mRNA marker LCE1C	RT-PCR	The expression levels of *LCE1C* decreased with increasing the time interval in a time-dependent manner, whereas no significant influences by temperatures’ changes	Ali M et al. [14]	28,796,650

**Table 2:** Studies on the tissue specificity of the epigenetic modifications in body fluids and post-mortem tissues

Tissues and organism	Epigenetic mark	Methods	Findings	Authors	PMID
Blood, saliva and vaginal secretions from 96 healthy Korean volunteers	DNA methylation	Illumina HumanMethylation 450 K bead array and pyrosequencing	Identified body fluid-specific DNA methylation markers: for blood, cg06379435 and cg08792630; for saliva, cg26107890 and cg20691722; for vaginal secretions, cg01774894 and cg14991487; for semen, cg23521140 and cg17610929	Park et al. [15]	25,128,690
100 peripheral blood, 96 menstrual blood, 100 saliva, 55 vaginal fluid and 91 sperm samples	DNA methylation	Illumina HumanMethylation BeadChips 27 and 450 k	The analysis considered potential factors influencing DNA methylation and revealed differential DNA methylation in 150 candidate loci in venous blood, menstrual blood, vaginal fluid, saliva, and sperm. Nine of those loci selected as the most promising markers	Forat et al. [16]	26,829,227
42 body fluid samples including venous blood, saliva, semen, vaginal fluid and menstrual blood	DNA methylation	Illumina Infinium HumanMethylation450 BeadChip array	8 CpG sites were included in a multiplex assay that differentiated between blood, saliva, semen, and vaginal fluid. Successful methylation profiles in aged or mixed samples	Lee et al. [17]	25,796,047
12 blood, 12 saliva, 12 semen, 3 vaginal fluid, and 19 skin epidermis samples + replication in 461 additional samples including 49 blood, 52 saliva, 34 semen, 125 vaginal fluid, and 201 menstrual blood samples	DNA methylation	Targeted bisulfite sequencing	Starting from 15CpG marker candidates, positive identification was obtained for blood, saliva, semen, vaginal fluid, and menstrual blood using the 9 CpG markers which showed a methylation signal only in the target body fluids	Lee et al. [18]	27,344,518
Blood samples and buccal swab from 55 healthy donor	CpGs in *PDE4C*, *ASPA*, *ITGA2B*, *CD6*, and *SERPINB5* genes	Pyrosequencing	A CpG in *PDE4C* identified for age-prediction and DNA methylation at 2 cell type–specific CpGs (in the *CD6* and *SERPINB5* genes) precisely discriminated the cellular composition in buccal swab samples and improved epigenetic age predictions based on other CpG sites	Eipel et al. [19]	27,249,102
23 venous blood samples, 24 buccal swabs, 22 vaginal secretions, and 20 semen samples from unrelated volounteers	DNA methylation	Pyrosequencing and quantitative PCR/high resolution melt analysis	Two markers, NMUR2 and UBE2U, were found to be specific for sperm, one marker (SA-6) found hypermethylated in saliva when compared to other body fluids	Alghanim et al. [20]	32,891,597
Brain specimens collected at autopsy from 7 subjects within 24 h after death	DNA methylation	Infinium HM450K array and Enhanced Reduced Representation Bisulphite Sequencing (ERRBS)	Numerous CpG sites identified are differentially methylated between GABAergic and glutamatergic neurons: greater number of undermethylated CpG sites in glutamatergic versus GABAergic neurons	Kozlenkov et al. [21]	26,612,861
Data from 3 individuals for 5 different regions of the cortex, the cerebellum, and pre-mortem blood	Mitochondrial DNA methylation	MeDIP-sequencing (Illumina Hi-Seq platform)	mtDNA methylation patterns found brain region specific and able to separate individuals belonging to the three main tissue types, blood, cortex, and cerebellum, based on mtDNA methylation variation	Devall et al. [22]	28,473,874
11 somatic postmortem tissues: cerebral cortex, spleen, heart, kidney, lung, mucosa from esophagus, stomach, pancreas, liver, bladder, and colon, from 6 individuals	DNA methylation, H3K4me3, H3K27me3	Illumina GoldenGate BeadArray genome-wide	DNA methylation patterns were largely conserved across 11 different tissues and across six individuals: similar levels in all organs and individuals for many CpG sites. DNA was highly methylated in non-CpG islands and/or CpG sites that are not occupied by either H3K4me3 or H3K27me3	Byun et al. [23]	19,776,032
Blood, saliva, semen, and vaginal secretion from 60 healthy volunteers	1700 miRNA	Genome-wide miRNA microarray	Forensically relevant miRNA markers identified: miR484 and miR182 for blood, miR223 and miR145 for saliva, miR2392 and miR3197 for semen, and miR1260b and miR654-5p for vaginal secretions	Park et al. [24]	24,915,788
Blood, semen, vaginal secretions, and menstrual blood from volunteers	452 human miRNA tested	Real-time quantitative PCR	Nine miRNA (miR451, miR16, miR135b, miR10b, miR658, miR205, miR124a, miR372, and miR412) were differentially expressed allowing the identification of the body fluid origin	Hanson et al. [25]	19,454,234
10 venous blood, 10 menstrual blood, 10 semen, 10 saliva, and 10 vaginal material samples from volunteers	miR451, miR412, miR891a, miR205, and miR124a	Real-time quantitative PCR	MiR451 confirmed as a biomarker for venous blood, miR412 for menstrual blood, and miR891a for semen. MiR205 was able to differentiate between saliva and semen and miR124a to differentiate between vaginal material and saliva	O Leary et al. [26]	29,714,155
200 samples, blood samples (peripheral blood and menstrual blood), and non-blood samples (saliva, semen, and vaginal secretion) from unrelated volunteers	miR451, miR205, miR214, and miR203	Real-time quantitative PCR and Fisher’s discriminant function	The expression of miR451 was significantly higher in the blood samples than in the non-blood samples. miR203, miR205, and miR214 allowed to distinguish menstrual blood from peripheral blood	He et al. [27]	31,734,726
605 body fluid–related samples from volunteers, including 136 peripheral blood, 102 menstrual blood, 129 saliva, 114 semen, and 124 vaginal secretion samples	10 body fluid–specific miRNA markers (miR451a, miR205-5p, miR203a-3p, miR214-3p, miR144-3p, miR144-5p, miR654-5p, miR888-5p, miR891a-5p, and miR124-3p)	Real-time quantitative PCR	A cluster of 4 miRNA (miR451a/miR891a-5p/miR144-5p/miR203a-3p) completely identify the peripheral blood, menstrual blood, and semen	Liu et al. [28]	33,313,714
61 tissue biopsies of 24 different organs from 2 male bodies	2007 miRNA of miRBase V19 (first corpse) and 1997 human miRNA of miRBAse v21 (second corpse)	SurePrint 8 × 60 K Human V19 miRNA microarrays (Agilent) and tissue specificity index calculation	Tissue-specific miRNA identified: miR122-5p, miR7-5p, and miR205-5p for liver, pituitary gland, and skin, respectively; a muscle cluster with miR133b, miR133a-3p, miR1-3p; a brain-tissues cluster including mi338-3p, miR219a-5p, miR124-3p, miR9-5p, and miR507; testis cluster with miR514a-3p and miR509-5p	Ludwig et al. [10]	26,921,406
Blood samples from three sites (peripheral blood, the inferior vena cava for pre-cardiac blood, and the coronary sinus for post-cardiac blood) collected from 28 forensic autopsy cases	miR39, miR208b, miR1, miR16, let-7e	Real-time quantitative PCR	Cardiac miRNA levels, in particular miR208b and miR1, in postmortem cardiac blood are different according to three sampling sites. MiR16 and let-7e, the non-cardiac microRNA, do not show any significant quantitative differences between the sampling sites	Kim et al. [29]	33,516,145

From the literature analysis, a restricted number of studies emerged related to human samples concerning epigenetic modifications stability, compared to experiments involving animals. In addition, only a few human studies on tissue specificity, which analyzed mainly DNA methylation and miRNA, considered post-mortem samples. For this reason, several studies that used body fluids from living subjects as starting material were included to represent how epigenetic modifications have been studied in the forensic field.

The term “tissues” in the text refers to any biological sources, including body fluids.

## Results

Given the potential importance of epigenetics in the forensic research field, the epigenetic modifications, their functions, and potential applications in forensic science are described. The epigenetic approach might result in being superior to histological and immunological assays in certain forensic applications [21]. In addition, the literature related to confounders that should be considered in forensic epigenetic research, in particular, the post-mortem stability of the epigenetic modifications in cadaveric samples and their tissue specificity, has been analyzed. In fact, post-mortem tissues are frequently used with no clear understanding of the effect that post-mortem tissue decay could have on the epigenetic marks. Pathological antemortem conditions and the cause of death could deeply affect post-mortem changes, and they are hardly reproducible in animal experimental settings. Human post-mortem tissues offer the possibility to gain a direct understanding of the mechanisms of disease, overcoming the issues in the interpretation of the results, the reproducibility and reliability, and the lack of concordance [22, 23]. Due to the importance of working with human samples, we report some of the experiments performed to explore the stability of the epigenetic modifications in post-mortem human tissues (Table 1).

Moreover, tissue-specificity can be used to trace the tissue of origin at crime scenes. On the other hand, the tissue specificity of epigenetic marks can also be crucial in forensic pathology for analyzing cadavers to identify possible environmental exposures, diseases, causes of death, and more. However, in this context, it should be considered that epigenetic changes are tissue-specific, and tissue-specific patterns of gene expression often contribute to maintain tissue identity and function [24, 25]. Forensic scientists should be aware of these potential confounders before using epigenetic markers because they might bias the results. Studies in humans on the tissue-specificity of epigenetic modifications in post-mortem samples are lacking. We thus also described studies related to the tissue-specificity of epigenetic modifications in body fluids from living subjects (Table 2).

### DNA methylation and possible applications in forensics

DNA methylation can be considered the first level of epigenetic modification. Despite it was identified back in 1948 [26], its biological role in the regulation of transcription was demonstrated only 25 years later [27]. DNA methylation is the addition of a methyl group (-CH3) to the fifth carbon of the cytosine to form 5-methylcytosine (5mC), resulting in gene expression silencing. This change occurs mainly, but not exclusively, at the CpG dinucleotides [28]. CpG dinucleotides are not uniformly distributed throughout the genome with stretches of DNA, called CpG islands or CGi, characterized by a higher CpG density [29, 30]. CGi are often localized in genes’ promoters and usually not methylated. Conversely, CGi associated with intra- or inter-genic regions can be methylated or not methylated [31, 32]. This leads to a heterogeneous epigenetic landscape.

For several years, CGi in the regulatory genes’ regions were not thought to have a tissue-specific profile; however, more recently, it has been demonstrated that tissue-specific gene silencing for some genes occurs through the promoter region’s methylation [33]. Moreover, intragenic CGi and those including the transcription start site can be differentially methylated based on the analyzed tissue [34].

In addition to its central role in transcriptional regulation, DNA methylation is extremely important for the maintenance of cellular functionality and genomic integrity, silencing cryptic promoters and cryptic splicing sites, and thus preventing the production of proteins with abnormal function [35]. DNA methylation also helps maintain in a compact chromatin state the repetitive DNA sequences, as transposable elements and satellite DNA, ensuring genomic integrity and avoiding illegitimate recombination [36]. More generally, even if the precise mechanism has not yet been clarified, methyl groups directly contribute to generating a close chromatin structure and thus in its three-dimensional modeling [37, 38].

The enzymes carrying out DNA methylation are the DNA methyltransferases (DNMT). DNMT3a and DNMT3b are de novo methyltransferases, which act on not methylated sequences and mainly in germ cells where they are recruited on chromatin by DNMT3l [28]. DNMT1 is the maintenance DNA methyltransferase that is required to methylate the hemimethylated strand after DNA replication, and its action guarantees the mitotic inheritance of the methylation patterns [39]. The methylation profile is thus preserved during mitosis, especially in the differentially methylated sequences subjected to imprinting or the ones in the inactive X chromosome in females. By contrast, the methylation profile of sequences without precise regulative functions may not be accurately reproduced [40].

In mammalian cells, DNA methylation signals are recognized by the methyl-CpG binding proteins (MBP) that in turn recruit other partners, among which many enzymes involved in histone modifications and transcription repressive molecules. They thus provide the link between modified cytosines and functional chromatin states [41].

DNA methylation, like all the other epigenetic markers, is reversible [42]. Two mechanisms can remove this signature: active DNA demethylation mediated by the ten-eleven translocation (TET) family enzymes and the passive DNA demethylation occurring during DNA replication in the absence of DNA methylation maintenance activity [43].

Forensic scientists have proposed the analysis of differentially methylated regions in multiple applications [44]. First, age prediction is particularly relevant to narrow the circle of suspects during investigations, and it is possible thanks to the fact that DNA methylation patterns change with increasing age [45]. Initially, common methylation changes were detected in cancer and aging cells [46]. Subsequent experiments support the use of a “DNA methylation clock” to estimate age with high accuracy [47, 48]. More recently, the investigation of the impact of biogeographic ancestry stands out as particularly important for DNA methylation-based age predictions, since prediction differences recently emerged comparing the Middle East and Central Europe population [49].

Different studies also developed DNA methylation–based approaches for tissue and body fluids identification. In particular, the main tissues considered are blood, urine, skin, sweat, saliva, semen, vaginal fluid, and menstrual blood. This can help in the reconstruction of a crime assist or sexual assault events [50]. Even if further investigations are needed, other applications are under consideration. Since it was shown that epigenetic differences can be detected in monozygotic twins [51], DNA methylation is emerging as relevant in monozygotic twin differentiation, which has always been a significant challenge in criminal investigations, and specific markers have been proposed [52]. In fact, personal health and lifestyle and exposure to various environments make methylation patterns unique for each individual. Moreover, in light of the monoallelic expression due to the genomic imprinting, the parental origin of an allele could be identified by analyzing regions that result differentially methylated in the maternal and paternal alleles [53]. This could be crucial in overcoming the significant limitations of STR profiling, which is only useful when individuals are genetically different.

More recently, few studies explored DNA methylation for potential smoking habit prediction, identifying CpGs correlated with daily cigarettes among smokers of varying levels [54, 55]. However, replication studies are needed to make DNA methylation analysis a routine test for assessing the smoking status of unknown individuals.

The interest in DNA methylation in forensic pathology research is sustained also by the observation that ante- and post-mortem DNA show similar methylation amounts and characteristics. Conversely, in cases of advanced decomposition, the degradation of DNA will be reasonably followed by the concomitant loss of the corresponding methylation [56].

#### The post-mortem stability of DNA methylation

In animal models, it was demonstrated that the post-mortem interval (PMI) may represent a confounding factor in the analyses of 5-methylcytosine; in particular, 5-methylcytosine levels were observed to increase with post-mortem time in adult rats [57]. Changes in DNA methylation were also detected in human postmortem tissues: three studies explored DNA degradation and DNA methylation at multiple time points or stages of decomposition in different genome regions and tissues. Within PMI 38–68 h in humans, DNA samples collected from blood and brain were undamaged [58]. DNA yields were found high also in buccal swabs, and post-mortem methylation was stable from 1 up to 42 days. Even though it has been observed that both degradation and methylation variance increased over time [58], DNA methylation resulted stable also in human neocortex samples up to 72 h post-mortem [59]. Given that most forensic autopsies are performed within 72 h, it can be argued that many epigenetic markers can be studied in forensics without bias linked to the time elapsed since death (PMI). For post-mortem analyses that exceed this time range, further studies are required, and therefore, caution is needed.

#### Distinctive DNA methylation-based signatures across tissues

Since RNA is prone to degradation by ubiquitous ribonucleases, and thus its use for forensic identification of body fluids is very challenging [6], DNA methylation has been proposed as a new molecular marker for body fluid discrimination in the field of forensics. Specific CpG sites were identified with high sensitivity and specificity to discriminate between blood, saliva, semen, and vaginal secretions [60–65].

Interestingly, in light of the environmental impact on DNA methylation and for a more comprehensive simulation of forensic conditions, some of these studies analyzing body fluids from living subjects considered endogenous and exogenous factors that might affect the stability of methylation. Differentially methylated markers were detected for the identification of specific tissues, considering multiple influencing factors, such as humidity, tumors, genetic variants in the DNA sequence [61], or even the menstrual cycle phases, against which DNA methylation profiles can vary [63].

Other studies evidence the importance of considering confounding factors that may affect the results. For example, DNA methylation has been also proposed to estimate the age at the time of death in forensic profiling [66]; however, the different cellular compositions in the analyzed tissues should be well-known, such as buccal epithelial cells and leukocytes in buccal swab samples [64]. In the body fluid identification research, having the methylation patterns age-dependent [67], markers potentially associated with aging should be excluded [60].

Specific epigenetic patterns have been thus revealed especially in living subjects where it is often necessary to select the most useful surrogate tissue for representing the brain. The levels of DNA methylation in brain-peripheral tissues were shown to vary widely for each CpG and each gene, and tools were proposed to reveal the degree of cross-tissue correlation [68]. However, few studies determined inter- and intra-individual differences in DNA methylation in post-mortem tissues too. Kozlenkov and coworkers (2016) separated neuronal nuclei from the autopsy specimens of the human prefrontal cortex, evidencing differences in the composition of DNA methylation between the two major populations of human prefrontal cortex neuron subtypes, GABAergic interneurons and glutamatergic projection neurons [69].

The identification of blood, cerebellum, and the cortex from 3 individuals was also performed using tissue-specific patterns of mitochondrial DNA methylation. Mitochondrial DNA methylation variations among pre-mortem blood, post-mortem cerebellum, and 5 different regions of the cortex were able to separate individuals. Intra-individual differences across tissue types were greater than inter-individual differences within each tissue type [70].

Conflicting results were also evidenced. The analysis of 1505 CpG loci in 11 human tissues from six autopsy cases, in particular DNA methylation of 1505 CpG promoter sites in 807 genes, revealed that similar DNA methylation levels in all organs and individuals for many CpG sites were detected among the same tissues from different individuals than between different tissues from the same individual, but in general, the patterns were very homogenous. However, these results could be affected by the high variability of periods between death and the tissue collection and the diagnosis of autopsy [71]. Another research explored the reliability of 11 tissue-specific DNA methylation sites for the identification of blood, saliva, and semen; however, tissue-specific differentially methylated regions for blood and buccal cells were not specific enough to be suggested as markers for blood and saliva [72].

Even if some studies highlighted the existence of an inter-individual variation in the methylation levels [73, 74], recent evidence reported specific methylation patterns in different cell types [75] supporting the possibility of using this signature for forensic applications.

### Histone modifications and possible applications in forensics

Nuclear DNA is not naked within cells but is associated with proteins, mainly histones, to form chromatin. In 1964, it was discovered that histones might be subjected to post-translational modifications that confer important functional properties and affect the degree of chromatin condensation [76]. The most studied histone modifications are acetylation, associated with transcriptional activation, and histone methylation, associated with both transcriptional activation (H3K4) and repression (H3K9, H3K27). Many enzymes drive the addition and the removal of these modifications that are then recognized by different effector proteins [77].

It should be noted that on each histone, the coexistence of multiple signals might interfere, cooperate with, or be dependent on each other. Therefore, in each nucleosome, a huge number of combinations of different histone changes generate the histone code [78], suggesting the existence of a highly complex regulation system, still largely unknown [79]. The crosstalk takes place not only between different histone modifications but also between histone modifications and DNA methylation, which thus work together to modulate DNA accessibility through changes in the chromatin conformation [80].

The state of chromatin within a cell that swings between an open and accessible state named euchromatin and a more compact state, not accessible to the transcriptional machinery, defined as heterochromatin [81], might be modified by chromatin remodeler enzymes [82]. Chromatin remodeling is a dynamic event, not yet fully characterized, that requires ATP hydrolysis and plays a role in DNA replication, transcriptional regulation, and DNA repair through several mechanisms, such as nucleosome positioning, histone substitution, deposition of histone variant, chromatin compaction, and changes in its accessibility [82–84].

More recently, increasing evidence suggests that DNA is non-randomly positioned into the nucleus, with chromosomes, gene loci, and nuclear bodies referring to a specific arrangement in space and time [85]. This organization in precise sub-compartments might be the basis for regulating chromatin state and ensuring optimal transcriptional efficiency. However, how chromosome positioning could affect genomic function is not yet understood [86].

The histone modification profile has been less explored in comparison with the other epigenetic signatures in the forensic context. Few studies explored the stability of acetylation and methylation in post-mortem brain specimens, in particular H3K27Ac and H3K4me3 [16], and, investigating the genome-wide distribution of histone modifications in specific neuronal cells, the existence of distinctive patterns of the histone modifications was evidenced [87]. One of the current challenges in epigenetics is to analyze histone modifications and their differences among biological conditions and cell types. Considering the long and complex pipeline of the histone changes’ detection techniques, this analysis is currently difficult to apply in forensic practice. Improved experimental methods could reveal histones’ utility in forensic applications, as markers for the determination of the cause of death and tissue specificity. Furthermore, forensic sample handling processes typically do not preserve proteins such as histones. Despite the advancements in forensic protein science and technology in recent years [88], validation studies involving real-life applications are necessary before forensic proteomics, including histone analyses, can become a routine tool for deciphering crime scenes.

#### The post-mortem stability of histone modifications

Research investigated the post-mortem stability of histone modifications and related enzymes. The pattern of post-mortem degradation for H3K27 methylation and acetylation was reported in Sprague–Dawley rats, recording less stability of these modifications to histone tails over PMI, in comparison to other epigenetic signatures [89]. Huang and coworkers (2006) first evaluated chromatin degradation in post-mortem prefrontal cortex samples from 16 adult subjects, examining DNA-histone interactions with micrococcal nuclease digestion. The nucleosome DNA resulted attached to the core histones for 30 h after death, and differences in the levels of the open chromatin mark H3K4me3 and the condensed chromatin mark H3K27me3 across some genomic loci were similar to freshly prepared samples. In addition, the level of the H3 methylation seemed not highly affected by autolysis time (PMI range = 5–30 h) and pH variation (pH range = 6–6.8) [90]. The stability of H3K4me3 in the human prefrontal cortex was also confirmed up to 72 h post-mortem in another study that tested this epigenetic signature as a marker of methamphetamine use disorder in HIV-infected individuals [16]. These studies seem to point towards histone modifications as an alternative for transcriptional profiling in case of low RNA quality.

Another research highlighted that the acetyltransferase and methyltransferase activities, which are involved in the process of histone acetylation and methylation, were not modified increasing the PMI or storage duration. The same study also reported no influence of PMI of 5 h, storage, pH value, or neurochemical parameters on RNA integrity [91]. Of course, more studies exploring longer PMIs are requested to confirm the stability after death of histone modifications.

### Non-coding RNA and possible applications in forensics

The term non-coding RNA (ncRNA) refers to RNA not translated into proteins; however, this does not imply they do not have a specific function or carry information. The best-known and most studied ncRNA are structural ncRNA, which include RNA transfer (tRNA), ribosomal RNA (rRNA), small nuclear RNA (snRNA), and small nucleolar RNA (snoRNA). The remaining ncRNA are able to modulate gene expression and induce chromatin remodeling without affecting DNA sequence; therefore, they are considered a full-fledged epigenetic mechanism. Among these molecules, there are the long non-coding RNA (lncRNA) and the small non-coding RNA (sncRNA) among which microRNA (miRNA) [92].

The lncRNA class, constituted by transcripts not translated into proteins longer than 200 base pairs (bp), is highly heterogeneous. LncRNA can have multiple functions, among which the regulation of transcription, proteins/RNA functioning, chromatin remodeling, and the genome 3D organization in the nucleus [93].

MiRNA act through RNA interference (RNAi), a process that involves the RNA-induced silencing complexes (RISC) which incorporate miRNA and guide it towards a target mRNA thanks to the sequence complementarity [94]. In this way, RISC induces the mRNA degradation or its translational repression, depending on the full or partial complementarity with the miRNA sequence [95]. Although miRNA act at the post-transcriptional level and not transcriptional, they are accounted among the epigenetic mechanisms because they are involved in a complex regulatory epigenetic network. In addition, miRNA expression can be controlled by epigenetic modifications within the genome regions where they are located, and in turn, they can target epigenetic modifiers [96].

Even if miRNA biogenesis is well-known, the complex regulatory circuit underlying miRNA expression remains unclear. This landscape becomes even more complicated by the low specificity between miRNA and mRNA, which results in a variety of targets [97]. Subsequently, a single miRNA can regulate several targets, and the same targets can be co-regulated by different miRNA, originating a complex combinatorial code. More than 2600 miRNA (miRBase v.22) have been identified in humans, and they are involved in gene regulation mechanisms, metabolic pathways of development and differentiation, cellular interactions, and disease development [98].

Among the various applications of miRNA described in forensic medicine [99], there is tissue identification from a single source of body fluid, since they are less susceptible to degradation, due to their small length of 22 nucleotides on average, compared to mRNA [100]. In addition, in light of their stability, even in the face of temperature and environmental conditions changes, they have been identified as a valuable tool for estimating the post-mortem interval (PMI) [14]. Another application that should be further explored includes the determination of wound age, having miRNA involved in the wound healing progression [101]. Besides, their recognized role in myocardial infarction, due to their influence on cardiomyocyte regeneration, apoptosis, and necrosis, makes them a suitable marker for forensic studies on acute and chronic myocardial infarction, and on its timing [102].

Interestingly, studies also highlighted the potential of circular RNA (circRNA) in forensics, another class of single-stranded RNA molecules. CircRNA have been shown to regulate transcription and interact with miRNA and proteins [103]. In the forensic fields, due to their stage-specific expression patterns during development and stability, circRNA isolated from human blood were recently proposed for age prediction [104]. A preliminary model was developed to investigate potential associations between chronological age and the expression of circRNA derived from genes involved in biological metabolic processes. However, there is much to do to understand their function and mechanisms of action. For instance, although circRNA were originally classified as non-coding RNA, there is evidence of their involvement in translation processes [105]. This enhances the interest in circRNA in the context of forensic applications.

#### The post-mortem stability of miRNA

MiRNA are more resistant in various relevant clinical and research conditions [106] compared to longer RNA molecules, like mRNA. We already explained their role as epigenetic modulators, affecting the protein levels of the target mRNA without modifying the DNA sequences [107], and thus, we also report some studies exploring their stability after death and long-term fixation. Animal studies reported that miRNA were found highly resistant to PMI. A significant correlation was observed between miRNA expressions and time passed since death, with miR21 and miR205 stably expressed especially at 24 h PMI duration [108]. Extreme robustness across increasing PMIs, for up to 96 h, was shown for miR16, miR34a, miR124a, and miR134 [89].

Since, in forensic cases, fresh or frozen human material is not always available, studies analyzed formalin-fixed paraffin-embedded samples, highlighting sensitive forensic markers, even when signs of putrefaction were detected at autopsy. MiR21 resulted in a valid detectable molecular target in multiple post-mortem samples and putrefied organs considered at 18, 72, and 96 h of PMIs [109]. Comparing samples frozen or embedded in paraffin, the expression of miR146a, miR146b, miR125a, miR125b, miR21, and miR155 was identified as a signature of injured skin [110]; miR499a was confirmed as a promising acute myocardial infarction biomarker [111]. From those studies emerged also the importance of identifying endogenous controls for miRNA quantification, supporting the use of miRNA instead of other classes of small ncRNA to determine the appropriate controls for the post-mortem analysis [111].

The use of miRNA in forensic sciences has been widely explored in particular for PMI estimation. The duration of the corpse’s storage was reported to not affect the overall miRNA pattern of expression in different tissues; in particular, a storage time between 1 and 14 days at 4 °C has a limited influence [112]. Montanari and coworkers (2021) reported the related literature, and they suggested miRNA use mainly as target markers for longer PMI evaluation, instead of early and medium PMI. They also evidenced the lack of human data that limits the forensic application of PMI estimation based on miRNA analysis [14]. These studies suggest to better explore miRNA reliability and utility as biomarkers in post-mortem examinations.

#### Tissue-specific miRNA-based signature

Considering the small size of miRNA molecules, 20–25 bases in length, and their strong tissue specificity, studies explored their possible use in the forensic field for the assay of different body fluids in often degraded or compromised samples [113]. Some studies explored large sets of miRNA, while others tried to replicate the results obtained in previous studies including a restricted number of targets and improving the methods and the conditions.

A genome-wide miRNA microarray tested approximately 1700 miRNA to identify 20 body fluid samples. Eight previously unreported miRNA were detected as relevant miRNA markers because of specific expression in one body fluid and high expression levels: miR484 and miR182 for blood, miR223 and miR145 for saliva, miR2392 and miR3197 for semen, and miR1260b and miR654-5p for vaginal secretions. Among the previously reported miRNA, a good body fluid–specific expression pattern was confirmed for miR126, miR106a, miR451, miR185, miR486, and miR20a for blood, miR203 and miR205 for saliva, even if they were also expressed in the semen, and miR891a as semen-specific [114].

Another research explored 452 human miRNA in 20 human tissues using real-time quantitative polymerase chain reaction (RT-qPCR): nine miRNA (miR451, miR16, miR135b, miR10b, miR658, miR205, miR124a, miR372, and miR412) were differentially expressed allowing the discrimination of the body fluid origin of forensic biological stains [115].

To test a small number of specific targets, studies used RT-qPCR. One of those confirmed miR451 as a biomarker for venous blood, miR412 for menstrual blood and miR891a for semen; miR205 was shown to differentiate between saliva and semen and miR124a to differentiate between vaginal material and saliva [116].

MiR451 was also tested in 200 samples from peripheral blood, menstrual blood, saliva, semen and vaginal secretion and showed significantly higher expression in the blood samples than in the non-blood samples. The same authors even suggested the use of miR203, miR205, and miR214 to be used to distinguish between peripheral blood and menstrual blood [117]. More recently, these and other popular miRNA were tested in combinations in 605 body fluid–related samples to increase the probability of the assumptions based on their detection. MiR451a, miR144-5p/3p, miR888-5p or miR891a-5p, miR203a-3p, miR205-5p, and miR124-3p were all able to distinguish between two tissues; however, they were only partially body fluid–specific. By contrast, a four-miRNA combination (miR451a/miR891a-5p/miR144-5p/miR203a-3p) completely identified the peripheral blood, menstrual blood, and semen [118].

In two studies, miRNA were tested in post-mortem tissues, offering a real-world scenario in which fresh biopsy material is not always available for miRNA isolation. A miRNA microarray analysis including 2000 miRNA among 61 tissue biopsies of 24 different organs from 2 male bodies revealed that 143 out of all miRNA were detected in all tissues; in detail, miR1-3p was the overall most tissue-specific, and it was highly expressed in muscle and myocardium. Single tissue–specific miRNA were miR122-5p, miR7-5p, and miR205-5p expressed in the liver, pituitary gland, and skin, respectively. In addition, hierarchical clustering revealed groups of miRNA with tissue-specific expression: a muscle cluster with miR133b, miR133a-3p, miR1-3p, a brain-tissue cluster including mi338-3p, miR219a-5p, miR124-3p, miR9-5p and miR507, and miR514a-3p and miR509-5p exclusively detected in the testis samples. The study also confirmed that inter-organism variability was significantly lower than inter-organ variability [112].

More recently, it was also evidenced that the miRNA levels in the same tissue might be different because of different sampling sites. In particular, cardiac-specific microRNA levels differed in venous blood obtained from the external iliac vein, the inferior vena cava, and coronary sinus [119].

### Linking epigenetic modifications to gene expression changes

It should be noted that many studies focused only on one of the epigenetic modifications, often hypothesizing connections with gene expression changes. However, epigenetics involves complex processes and thus transcriptional changes are not always linked. To avoid erroneous conclusions, changes in post-mortem gene expression should be taken into account in the experimental design. In order to analyze mRNA expression in association with the epigenetic changes identified or to use mRNA in forensic investigation, post-mortem mRNA degradation across diverse human tissues should be carefully considered. In fact, comparing fresh and post-mortem brain tissues, remarkable differences have been detected in the transcriptional levels with specific genes surprisingly stable in fresh tissues, while the results from human post-mortem brain studies were highly impacted by the PMI [120].

One research explored approximately 2000 post-mortem samples from 15 tissues of 316 donors with PMI ranging from 1 to 27 h using RNA sequencing. Different RNA degradation levels were associated with distinct PMI, different sites in the same tissue, and even different genes’ functions. This means that the time of sample collection should be always considered depending on the tissues and genes of interest [121]. However, another research, establishing high-quality RNA up to 23 years at − 80 °C, reported that PMI was not a predictor of RNA quality and suggested the RNA integrity number, the RIN value, a more critical indicator of the suitability of post-mortem tissues [122]. Other research has also encouraged the consideration of environmental conditions, such as storage temperature, before interpreting the results [123, 124]. In human experiments, these might represent confounding factors and should be reported.

## Discussion

Epigenetic modifications that occur before death can provide valuable insights into vital processes. The present review reveals how understanding the “epigenetic status” of cells in post-mortem samples enables forensic scientists to record what cells were prompted to do, capturing a snapshot of their condition at the time of death. Forensic pathology has acquired an unprecedented interest in epigenetics because it acts as a crucial communication system, fostering dialogue among cells in various tissues and organs, particularly during processes like inflammation resulting from physical or hypoxic-ischemic trauma [125].

This opens new possibilities for forensic researchers in the understanding of the physiopathology of the deceased, which can provide valuable information to the forensic pathologist, contributing to the understanding of the physiopathology of the living as well. Similarly, the relative resistance towards post-mortem phenomena found in various epigenetic markers [53] paves the way for numerous studies of what happens in the human body after death. This favors the potential for fitting the PMI estimation within increasingly specific ranges. In addition, epigenetic changes are tissue-specific, and tissue-specific patterns of gene expression often contribute to maintain tissue identity and function [71]: crime scenes and body investigations can greatly benefit from the ability of identifying various biological fluids precisely and specifically. Further research might help to gain better insight into the tissue-specificity of these markers.

Forensic cases thus encompass a wide range of conditions of interest to research, also including psychological trauma and physical injuries. This helps in a better understanding of human reactions to events. However, it is important to note that this is a potential future scenario. Currently, there are still numerous obstacles to overcome in order to obtain reliable and useful results in the context of trials.

The majority of the forensic epigenetic experiments focused on miRNA expression and DNA methylation. The methods used are mainly array-based experiments for genome-wide approaches, to test simultaneously a wide range of sites, and pyrosequencing for candidate region association studies. Differentially methylated cytosines and regions [38] and specific miRNA have been demonstrated to differ between tissues and body fluids relevant in forensic analyses. A highly intriguing result is that four miRNA (miR205, miR451, miR124, and miR203) are recurrent in multiple experiments. Three of these studies included a wide range of miRNA with microarray approaches, without prior hypotheses, but the identified miRNA often showed different body fluid–specific expression patterns. For example, miR205 was identified as a marker for saliva [60, 115]; however, Ludwig and coworkers reported a highly specific expression of miR205-5p in the skin [112], and it was recently reported for the identification of vaginal secretion [118]. One notable finding from studies that have examined DNA methylation is instead that this mark is involved in the regulation of several molecular mechanisms; it is cell-specific, as showed by cell-specific differentially methylated regions identified in post-mortem brain areas [69, 126], and it is widely affected by environmental conditions throughout life. In particular, confounding factors, such as early life events [127], smoking, ethnicity, and gender [128, 129] and diseases, can modify DNA methylation levels at specific sites in the genome. Major application issues might thus arise for forensic pathologists in this case because the subjects’ history is not always known. In addition, studies also underline that part of the mechanism that causes post-mortem methylation levels to be modified or unmeasurable may involve reactive oxygen species, found increased over time [57], or post- or perimortem cellular processes or bacterial activity [56]. Even in gene expression studies, confounding factors should be taken into account, and correcting the results with knowledge about the cause and timing of death is often advisable, as ongoing changes prior to death could also play a significant role in the specific mRNA levels. Additionally, mRNA appears to be less stable and persistent compared to other markers under different environmental conditions [130].

### Limitations of the studies

In general, studies reveal some weaknesses; first, often only one epigenetic mark and one type of specimen at a single time point were analyzed. To unlock the full potential of epigenetic testing for post-mortem applications, it is essential to include diverse tissues and apply diverse marker combinations at different stages of decomposition. Furthermore, due to the different identification methods, the techniques applied and normalization strategies, the results are not equivalent limiting their value for comparison. Due to the scarcity of available cases that satisfy the inclusion criteria, the studies often include an insufficient number of samples which does not allow to achieve solid conclusions. Two specific additional limitations arise from the analysis of miRNA and DNA methylation in the forensic field. First, not all the studies specified which of the mature forms of miRNA-3p and -5p were considered in the experiments, while the two forms might have different tissue specificities [112]. Second, not all the studies considered that DNA methylation is associated with aging [131]. Each experiment should subsequently exclude DNA methylation associated with aging from the analysis of tissue-specific DNA methylation candidates. As proposed by Park and coworkers (2014), the association between aging and possible DNA methylation changes in the analyzed genome sites should be investigated during the selection of these markers in forensic science [60].

Finally, it must always be noted that epigenetic modifications might play a central role in specific pathologies: specific miRNA, DNA methylation patterns, and histone changes are involved in carcinogenesis processes [132, 133] and have been found dysregulated in multiple cancers [134, 135], in neurodegenerative disorders [136, 137], in obesity and type 2 diabetes [138], and in cardiovascular disease [139]. This means that the physical and psychological conditions in which a person was prior to death might highly affect the level of expression of these molecules.

### Perspectives

Despite the limitations, and while many studies are performed on samples from living individuals, there are numerous advantages to working with and researching post-mortem samples. A more comprehensive epigenetic analysis of cells in post-mortem samples can offer new opportunities for identifying markers related to injuries, age prediction, the timing of events such as myocardial infarction or cerebral contusion, or the cause of death, whether due to asphyxia or hypoxic factors. These insights are significant in the field of forensic pathology. Compared to samples taken from living patients, human post-mortem tissues also provide the advantage of allowing the collection of larger amounts of starting material.

Another aspect to work on in the future concerns the fact that combinations of different miRNA, rather than individual molecules, might enable a more precise identification of body fluids. This is because single miRNA are only partially specific to particular body fluids, and their expression levels may not remain stable for a specific type of tissue. Just as in the case of forensic human identification through DNA profiling using autosomal short tandem repeats, which relies on the analysis of multiple loci, in the future, miRNA profiling for tissue specificity should consider clusters of miRNAs, as proposed by some studies [112, 118].

It should also be noted that epigenetic marks have been explored for a wide range of applications, including the identification of biological fluids or tissues, as well as the determination of sex, age, and phenotype of donors. Epigenetic modifications have been also associated with many pathological conditions and psychiatric diseases [123, 124]. However, given the substantial influence of environmental confounders on these marks, further research is needed to fully understand how to exploit the potential of epigenetics in revealing phenotypic and behavioral traits, thus expanding our comprehension of complex forensic evidence.

To promote the innovative use of epigenetic markers in forensic practice, studies should simultaneously investigate the differential expression of epigenetic phenomena in multiple tissues and organs. Implementing a strategy of multicenter studies, where large case datasets are analyzed with consistent inclusion/exclusion criteria and methods, could address the current limitations related to comparability among studies.

## Conclusions

Over the past decade, epigenetics has undergone rapid development, thus drawing attention to its potential applications in forensic investigations. In the present review, we described the limiting factors that should be taken into account in epigenetic research when applied in forensic medicine, including considerations of post-mortem stability and tissue specificity. While research in this field presents numerous potential forensic applications, it is important to exercise caution when applying these results in forensic cases. Forensic pathologists have long sought markers as incontrovertible evidence in trials [140]; however, these markers should be unquestionable and able to withstand criticism. Working synergistically on the perspectives, it may become possible to provide researchers with guidance on selecting epigenetic markers based on the available biological samples and techniques.
